# Nitazoxanide Exerts Immunomodulatory Effects on Peripheral Blood Mononuclear Cells from Type 2 Diabetes Patients

**DOI:** 10.3390/biom11121817

**Published:** 2021-12-02

**Authors:** Mauricio Castillo-Salazar, Fausto Sánchez-Muñoz, Rashidi Springall del Villar, Gabriel Navarrete-Vázquez, Adrián Hernández-DiazCouder, Carlos Mojica-Cardoso, Sara García-Jiménez, Cairo Toledano-Jaimes, Germán Bernal-Fernández

**Affiliations:** 1 Pharmacy Faculty, Universidad Autónoma del Estado de Morelos, Cuernavaca 62209, Mexico; mau.castillosalazar@gmail.com (M.C.-S.); gabriel_navarrete@uaem.mx (G.N.-V.); saragarcia@uaem.mx (S.G.-J.); tjcd_ff@uaem.mx (C.T.-J.); 2 Immunology Department, Instituto Nacional de Cardiología Ignacio Chávez, Tlalpan, Mexico City 14080, Mexico; fausto22@yahoo.com (F.S.-M.); raspringall@yahoo.com (R.S.d.V.); adrian.hernandez.diazc@hotmail.com (A.H.-D.); 3 Pathology Laboratory, Hospital del Niño Morelense, Cuernavaca 62275, Mexico; carlos.mojica.cardoso@gmail.com

**Keywords:** immunomodulation, nitazoxanide, inflammation, T2D, miRNAs

## Abstract

Background: Type 2 diabetes (T2D) is a low-grade inflammatory condition with abnormalities in the immune response mediated by T lymphocytes and macrophages. Drug repositioning for immunomodulatory molecules is an attractive proposal for treating T2D. Nitazoxanide (NTZ) is a broad-spectrum drug with promising immunomodulatory effects. Thus, we investigated the immunomodulatory effect of NTZ on peripheral blood mononuclear cells (PBMCs) from patients with T2D. Methods: Fifty patients with T2D were selected, and the proliferative response of T lymphocytes and the M1/M2 ratio of macrophages post cell culture were evaluated by flow cytometry, as well as measuring the concentration of cytokines by ELISA and the relative expression of microRNAs (miRNAs) related to the immune response by real-time PCR. Results: NTZ exerts an inhibitory effect on the cell proliferation of T lymphocytes stimulated with anti-CD3 and anti-CD28 antibodies without modifying cell viability, and significant decreases in the supernatant concentrations of interleukin (IL)-1β, IL-2, IL-6, IL-10, and IL-12. Furthermore, NTZ negatively regulates the relative expression of miR-155-5p without changes in miR-146a-5p. The M1/M2 ratio of monocytes/macrophages decreased the M1 and increased the M2 subpopulation by NTZ. Conclusions: Our results suggest that NTZ exerts immunomodulatory effects on PBMCs from T2D patients, and shows potential alternative therapeutic benefits.

## 1. Introduction

Type 2 diabetes (T2D) is a chronic degenerative inflammatory disorder characterized by insulin dysfunction and chronic hyperglycemia [1]. In 2019, the International Diabetes Federation (IDF) reported 463 million people with diabetes worldwide, with a prevalence of more than 9%, 1 in 2 cases of which had not yet been diagnosed, and it is projected that there will be 700 million cases in 2045. T2D represents 90% of all the cases of diabetes, and its inadequate control leads to cardiovascular disease, kidney failure, neuropathy, and lower limb amputation, resulting in frequent hospitalizations and premature death [2]. Although there are many pharmacological strategies for treating T2D, new therapies are still needed. Chronic systemic inflammation is recognized as a causative factor of the pathogenesis in T2D [3,4], producing abnormalities in the immune response [5], such as proliferation and activation of T lymphocytes and macrophages; functional deterioration of B lymphocytes and natural killer cells; and decreased chemotaxis, phagocytosis, and opsonization [6]. The activation of T cells plays a role in promoting and maintaining inflammatory processes [7]. Chronic hyperglycemia has been reported to deregulate the function of T cells [8], resulting in a significant loss to the natural regulatory mechanism mediated by T regulatory cells (Treg) in T2D, which further exacerbates the activation and inflammation for T cells [9]. Furthermore, an increase in proinflammatory cytokines is associated with poor glycemic control and disease complications [10]. It is widely accepted that macrophage activation is affected by T cells [11]. Patients with T2D have been reported to have a significant increase in M1 proinflammatory macrophages and a decrease in M2 anti-inflammatory macrophages [12,13]. M1 macrophages induce an inflammatory and insulin resistance state through the inhibition of insulin signaling, indirectly produced by TNF-α, IL-6, and IL-1β [14,15], while M2 macrophages protect against insulin resistance by an IL-10-dependent mechanism [16]. The cytokines secreted by immune cells are dominant regulators of pathological inflammation in T2D; therefore, macrophage signaling also affects polarization of the adaptive immune system to maintain a chronic T cell response [17]. Recently, miRNAs have been recognized as key regulators of the immune response, i.e., more than 100 different miRNAs are expressed by the cells of the immune system [18], which together influence the pathways that control cell development and function for innate and adaptive immune responses, which regulate cellular responses by targeting multiple components of a biological pathway [19,20]. The miR-155-5p and miR-146a-5p are transcriptionally regulated by NF-κB and have multiple functions. The expression of miR-155-5p has been shown to be essential for the development and function of T cells and macrophages, enhancing the inflammatory response, while miR-146a-5p functions as an anti-inflammatory regulator in various types of immune cells, suppressing the inflammatory process. These miRNAs both form a unique regulatory network to ensure precise inflammatory responses by regulating the activity of NF-κB, essential to control cell growth, proliferation, and differentiation, as well as the cytokine synthesis, whereas its dysregulation leads to the development of chronic inflammation in T2D [21,22]. Due to high failure rates, elevated costs, and the slowness in discovering and developing new drugs, the repositioning of existing drugs is an attractive proposal that involves the use of risk-free compounds, with lower overall development costs and shorter delivery timelines for the treatment of various types of diseases [23]. Nitazoxanide (NTZ) is a thiazolide-type drug synthesized by amidation from acetylsalicylic chloride acid and 2-amino-5-nitrothiazole with a broad spectrum of anti-infective effects that notably modulate the survival, growth, and proliferation of a variety of extracellular and intracellular protozoa, helminths, anaerobic, and microaerophilic bacteria, as well as viruses [24,25,26]. NTZ is rapidly hydrolyzed by esterases present in plasma to an active metabolite, i.e., tizoxanide via deacetylation. Tizoxanide is a very polar molecule with low cell permeability, which is the reason why NTZ is administered as its prodrug [27]. Moreover, a few studies have reported that NTZ has prominent immunomodulatory functions, exerting inhibitory effects in the production of IL-6 by RAW264.7 cells and lipopolysaccharide-stimulated peritoneal macrophages, indicating its regulatory role at the transcriptional level [28]. Previously, NTZ was proposed as an agonist of PPAR-γ receptors [29], as well as a cytokine inhibitor, emphasizing its potential role against the “cytokine storm” in COVID-19 [30]. Therefore, new approaches must be developed for the treatment of T2D that investigate alternative mechanisms for regulating important immune functions. NTZ is an interesting drug candidate for the treatment of meta-inflammation that characterizes T2D. Thus, the objective of this study was to evaluate the immunomodulatory effects of NTZ in cultured PBMCs from patients with T2D without complications, when stimulated with anti-CD3 and anti-CD28 antibodies.

## 2. Materials and Methods

### 2.1. Nitazoxanide Isolation from Commercial Tablets

Tablets containing 500 mg of NTZ were used. The covers were removed, and the tablet cores were gently disintegrated and reduced to powder using a mortar. NTZ was isolated from excipients using a heterogeneous extraction with acetone and filtration at room temperature, and NTZ was isolated from the mother liquor by evaporation, with a yield of 72%. The purity of the isolated NTZ was confirmed by melting point comparison of spectroscopic, spectrometric, and elemental analysis data. The melting point of the sample obtained experimentally was 202 °C, which correlated with that reported in the literature (202 °C) [31,32]. In addition, the nuclear magnetic resonance analysis of the isolated sample provided the following data: ^1^H NMR (400 MHz, DMSO-d6) d 2.18 (s, 3H, CH3); 7.27 (dd, 1H, H-3′); 7.60 (td, 1H, H-4′); 7.28–7.30 (m, 1H, H-5′); 8.16 (dd, 1H, H-6′); 8.74 (s, 1H, H-4); 10.81 (s, 1H, N-H). 13C NMR (100 MHz, DMSO-d6) d 20.9 (CH3), 117.8 (C-3′), 125.4 (C-5′), 131.4 (C-4′), 132.1 (C-6′), 132.9 (C-1′), 139.4 (C-5), 149.9 (C-2′), 153.2 (C-4), 162.3 (C-2), 163.2 (NHC=O), and 170.5 (OC=O) ppm. MS (FAB+): *m*/*z* 308 (M+H)^+^. The data collections matched with those reported in the literature [33]. The elemental analysis confirmed ≥95% sample purity (±0.3% of the calculated value): Anal. Calcd. for C12H9N3O5S: C, 43.90; H, 2.95; N, 13.67; S, 10.44. Found: C, 46.71; H, 2.94; N, 13.71; S, 10.39 [34].

### 2.2. Study Population

The sample size was obtained through a statistical adjustment, considering the design of the study. The calculations were carried out by means of statistical adjustments when determining variables and parameters previously evaluated by other research groups [35]; similar studies have used a sample size similar to that used in this study [36,37]. Fifty consecutive, randomized, and willing-to-participate patients with a clinical diagnosis of T2D without complications were invited to attend an external medical consultation at the Instituto Nacional de Cardiología “Ignacio Chávez” in Mexico City. Patients with a diagnosis of T2D equal to or less than ten years, with pharmacological treatment, and without complications were included in our study. Patients with infectious diseases, cancer, hemoglobinopathies, or advanced chronic complications were excluded. Sociodemographic, lifestyle, physical activity, and nutritional data were obtained using a questionnaire at the time of inclusion. The protocol was approved by the research and bioethics committees of the Instituto Nacional de Cardiología “Ignacio Chávez” (No. 20-1183). All participants signed an informed consent to participate.

### 2.3. Isolation and Culture of Peripheral Blood Mononuclear Cells (PBMCs)

After 8–10 h of fasting, for all patients, 15 mL of peripheral blood was obtained by venipuncture with EDTA collector tubes (Becton Dickinson, Franklin Lakes, NJ, USA). The HbA1c percentage was determined using a Cobas C111 analyzer (Roche, Basel, Switzerland). The PBMCs were obtained by centrifugation in Ficoll (Sigma-Aldrich, St. Louis, MO, USA) at 1500 rpm for 30 min. Phosphate-buffered saline (PBS) washes were performed, and viability was evaluated using trypan blue (0.04%) (Sigma-Aldrich, St. Louis, MO, USA). A total of 5 × 10^5^ PBMCs/well were cultured in sterile 24-well culture plates (clear, polystyrene, and flat bottom) (Corning, New York, NY, USA) with RPMI-1640 medium supplemented with L-glutamine, 1% of penicillin/streptomycin, and β-mercaptoethanol (Sigma-Aldrich, St. Louis, MO, USA) and 10% fetal bovine serum (Thermo Fisher Scientific, Rockford, IL, USA).

### 2.4. Cell Proliferation Assays

For the cell proliferation assays, 5 × 10^5^ PBMCs exposed to different treatments were labeled with CPDF-670 fluorophore (Cell Proliferation Dye eFluor, Thermo Fisher Scientific, Rockford, IL, USA), which was used to monitor individual cell divisions by binding to any cellular protein containing primary amines; then, the dye was equally distributed among daughter cells, measured as a successive halving of the fluorescence intensity of the dye up to 6 generations [38]. Cells without monoclonal antibody stimulation and without treatment with NTZ formed the control treatment; cells treated with 100 µM NTZ formed the NTZ treatment; cells stimulated with 100 ng/mL of anti-CD3 (mouse IgG2a, clone OKT3) (American Type Culture Collection, Manassas, VA, USA) and 500 ng/mL of anti-CD28 antibodies (mouse IgG1, clone CD28.2) (BioLegend, San Diego, CA, USA) formed the Abs treatment; cells stimulated with anti-CD3 and anti-CD28 antibodies treated with 100 µM NTZ formed the Abs + NTZ treatment.

The NTZ concentration was selected based on research by Seong Keung Hong et al. [28] and our previous standardization of the methodology, in which we found similar effects between NTZ 100 µM and higher concentrations. For the cell cultures, NTZ was dissolved as a 100 mM stock solution in dimethyl sulfoxide (DMSO), and titrations were made by 1:10 dilutions with PBS until reaching a concentration of 100 uM. The final concentration of DMSO in culture media was less than 0.1%. Once the PBMCs were cultured, the anti-CD3 and anti-CD28 antibodies used for the stimulation of T cells and NTZ 100 µM were added simultaneously. In the case of the different controls used in the study, only the NTZ or antibodies were added immediately after cultivating the PBMCs. Proliferation tests were performed in all the applied treatments. To ensure the activation of T cells through the stimulation system with anti-CD3 and anti-CD28 antibodies (4 days or more) [39,40], and because miRNAs involved in an immune response are differentially expressed after T cell stimulation (3–7 days) [41], we selected 120 h of cell culture at 37 °C and 5% carbon dioxide (CO_2_) in a humid atmosphere. The samples were measured and quantified by flow cytometry (FACS Aria, BD Biosciences, Franklin Lakes, NJ, USA) through the incorporation of the fluorophore CPDF-670. The cell viability assay was performed by staining and counting live or dead cells with trypan blue dye (0.04%) to evaluate the integrity of the cell membrane.

### 2.5. Cytokine Levels in Supernatants of Cell Cultures

Supernatants of all treated cell cultures were collected and concentrations of IL-1β (900-K95), IL-2 (900-K12), IL-4 (900-K14), IL-6 (900-K16), IL-10 (900-K21), and IL-12 (900-K96) were determined by enzyme-linked immunosorbent assays (ELISAs) using commercial kits (PeproTech, Inc., Cranbury, NJ, USA), following the manufacturer’s instructions.

### 2.6. RNA Isolation from PBMCs and miRNA Determination by RT-qPCR

The total RNA was extracted from PBMCs by the QIAzol method, following the manufacturer’s protocol (QIAGEN, Hilden, Germany). The RNA concentration and purity were evaluated using a Nanodrop 1000 (Thermo Fisher Scientific, Rockford, IL, USA). The miR-155-5p and miR-146a-5p expressions were determined using an RT primer assay and hydrolysis probes (Applied Biosystems, CA, USA). The miRNAs were determined using a two-step RT-qPCR with an RT primer specific assay in combination with TaqMan probes (Applied Biosystems, CA, USA). Each RT reaction used 2.5 µL (30 ng of RNA) from the 14 µL eluted RNA using a TaqMan MicroRNA Reverse Transcription Kit (Applied Biosystems, CA, USA). The miRNAs were detected and quantified using miRNA assays for miR-155-5p (assay ID 463509_mat, Applied Biosystems, CA, USA), miR-146a-5p (assay ID 001187, Applied Biosystems, CA, USA), and U6 as reference gene (assay ID 001973, Applied Biosystems, CA, USA); 1.5 µL of RT reaction was amplified in 10 µL reactions. PCR was performed using a LightCycler TM 480 II System (Roche Applied Science, Basel, Switzerland) with a LightCycler 480 Probes Master kit (Roche Applied Science). The miRNA relative concentrations were normalized with the Ct values of U6, and values were calculated using the 2^−ΔΔCt^ method.

### 2.7. Determination of M1 and M2 Monocyte/Macrophage Subpopulations by Flow Cytometry

For each patient, 1 × 10^5^ of antibody-stimulated PBMCs (Abs), as well as those stimulated cells treated with NTZ (Abs NTZ) were cultured under the same conditions. After 120 h, cells were harvested and washed with supplemented PBS with 1% fetal bovine serum (FBS) (Thermo Fisher Scientific, Rockford, IL, USA) and 0.1% sodium azide (Sigma-Aldrich, St. Louis, MO, USA) and then stained with PE-Cy7 Mouse Anti-Human CD-14 (#557742), FITC Mouse Anti-Human CD-16 (#555406), and PE Mouse Anti-Human CD-163 (#556018) monoclonal antibodies (Becton Dickinson, Franklin Lakes, NJ, USA). The expression of CD-163 was used to determine the presence of M2 or M1 populations. Unlabeled cells and isotype control antibodies were used as references (controls). Cell subpopulations were quantified on an FACS Aria cytometer (BD Biosciences), using FACS Diva software (BD Biosciences) and FlowJo 7.6.2 program (Three Star, Ashland, OR, USA).

### 2.8. Statistical Analysis

Data are expressed as median. Friedman’s test followed by a Tukey’s test were used for multiple sample analysis. Correlations between variables were determined by Pearson’s test. *p* < 0.05 was considered statistically significant. Statistical analyzes were performed with the GraphPad Prism v7.0 (GraphPad Inc., La Jolla, CA, USA) and SPSS v15.0 software (SPSS Inc., Chicago, IL, USA).

## 3. Results

### 3.1. Description of the Population

The study population consisted of 38 women (76%) and 12 men (24%), with a median age of 53 years. According to the self-report questionnaire, the referred patients presented with the following: hypertension (56%), heart disease (20%), alcohol consumption (less than 21 standard drink units/week) (24%), frequent consumption of sugar and/or soft drinks (1000 mL minimum weekly) (50%), smoking (12%), moderate physical activity (3 to 6 MET) (58%), balanced diet (consistent 2000 kcal/day intake) (56%), and metformin as an antihyperglycemic treatment (84%).

At the blood sample collection, 45 (90%) patients (35 women and 10 men) were overweight or obese (body mass index ≥ 25.0 kg/m^2^) and 43 (86%) patients (35 women and 8 men) had an increased waist-to-hip ratio (>0.84 for women and >0.94 for men). According to the percentage of HbA1c and the American Diabetes Association (ADA) guidelines, 15 (30%) patients were controlled, and 35 (70%) patients were uncontrolled, with an average of 5.49% and 10.15%, respectively.

### 3.2. NTZ Inhibits CD3- and CD28-Induced T Cell Proliferation

**Figure 1:**
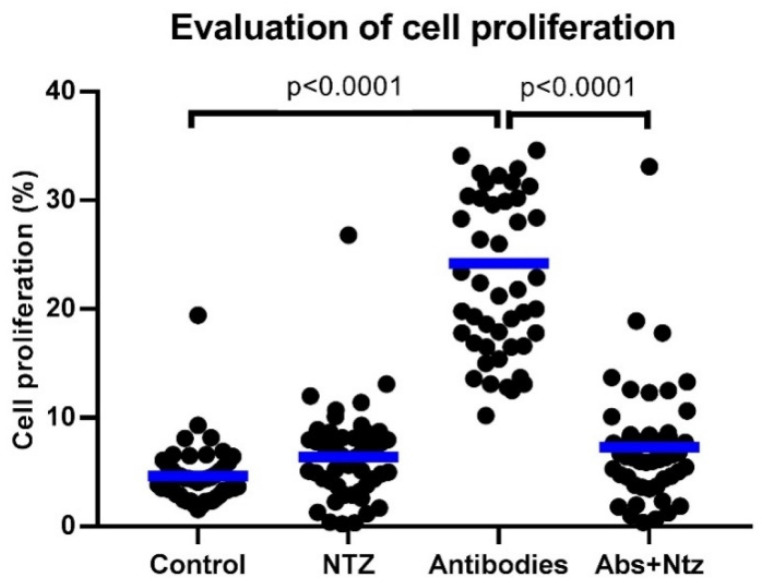
NTZ inhibits cell proliferation induced by anti-CD3 and anti-CD28 in PBMCs. Cell proliferation was evaluated after 5 days using CPDF-670 in PBMCs with RPMI-1640 cell media (Control), treated with NT Z100 µM (NTZ), stimulated with anti-CD3 and anti-CD28 antibodies (Antibodies), and antibodies plus NTZ (Abs + NTZ). The scattered dot plot shows individual measurements, and the bar represents the median. Friedman test for paired samples *p* < 0.05, Tukey’s DHS post hoc, and exact *p* values for individual tests are shown.

As shown in Figure 1, the percentage of cell proliferation of antibody-stimulated cells treated with NTZ (Abs + NTZ) was three times lower as compared with the percentage found in the antibody-stimulated cells (antibodies) (*p* < 0.0001) and was similar to the control group.

**Figure 2:**
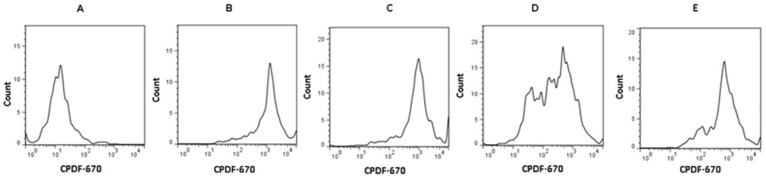
NTZ suppresses T cell proliferation. Histograms of flow cytometry analyses showing CPDF-670 unlabeled cells cultured in the absence of stimulus or treatments (**A**), CPDF-670 labeled PBMCs cultured without stimulus or treatments (**B**), following CPDF-670 labeled PBMCs with treatment NTZ 100 µM (**C**), CPDF-670 labeled PBMCs and stimulated with anti-CD3 and anti-CD28 (**D**), and CPDF-670 labeled PBMCs with CPDF-670 and PBMCs stimulated with antibodies anti-CD3, anti-CD28 and treatment with NTZ 100 μM (**E**).

Furthermore, cell viability was similar in all the evaluated conditions: control 91%, antibodies 95%, NTZ 87%, and Abs + NTZ 89%. Figure 2 shows the histograms of cell proliferation obtained by flow cytometry.

### 3.3. NTZ Lowers Proinflammatory Cytokines in PBMC Supernatants

**Figure 3:**
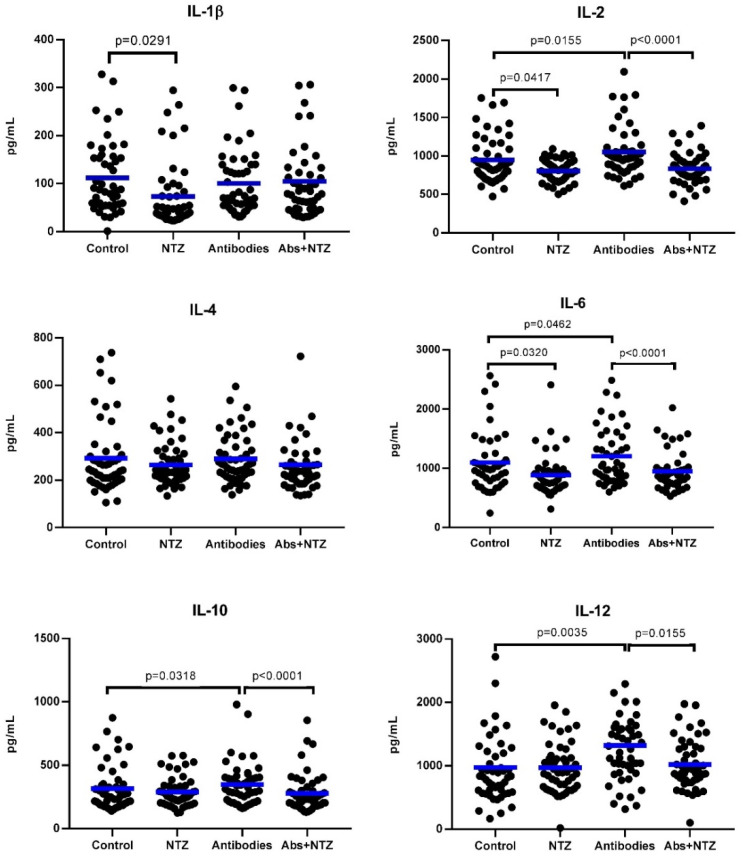
NTZ reduces levels of IL-1β, IL-2, IL-6, IL-10, and IL-12 cytokines in the PBMC cell supernatant. The measurement was made after 5 days of cell culture using the ELISA assay, and the cytokines were evaluated according to the type of treatment carried out on the PBMCs: cells with RPMI-1640 cell medium (Control), treated with NTZ 100 µM (NTZ), stimulated with anti-CD3 and anti-CD28 antibodies (Antibodies), and antibodies plus NTZ (Abs + NTZ). The scattered dot plot shows individual measurements, and the bar represents the median. Friedman’s test for paired samples *p* < 0.05, Tukey’s DHS post hoc, and exact *p* values for individual tests are shown.

As compared with the cytokine levels produced by cells without treatment (control), the cells treated with NTZ showed significant decreases in IL-1β (*p* = 0.0291), IL-2 (*p* = 0.0417), and IL-6 (*p* = 0.0320) concentrations, while cells stimulated with antibodies showed significant increases in IL-2 (*p* = 0.0155), IL-6 (*p* = 0.0462), IL-10 (*p* = 0.0318), and IL-12 (*p* = 0.0035) concentrations. However, those increments were inhibited when antibody-stimulated cells were treated with NTZ (IL-2, *p* < 0.0001; IL-6, *p* < 0.0001; IL-10, *p* < 0.0001; and IL-12, *p* = 0.0155), re-establishing the concentration levels to those similarly detected in cells treated with only NTZ, demonstrating the inhibitory effects of NTZ on the production of cytokines induced by antibodies in PBMCs. Interestingly, concentrations of IL-4 were not altered by any treatment, while IL-1β concentration levels were decreased with NTZ (*p* = 0.0291) and did not show changes when treated with antibodies or Abs + NTZ (Figure 3).

### 3.4. NTZ Downregulates miR-155-5p Expression in PBMCs

**Figure 4:**
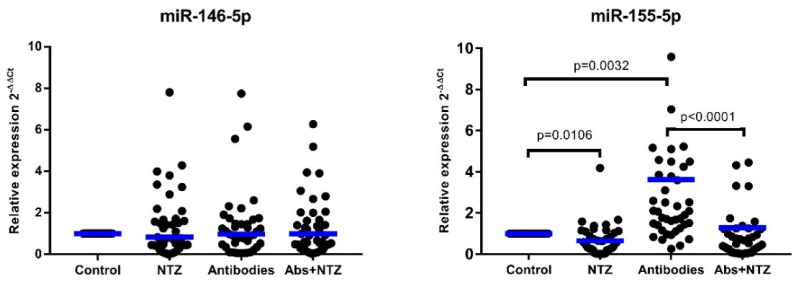
NTZ regulates the expression of miR-155-5p without causing changes in the expression of miR-146a-5p in PBMCs. Total RNA was extracted from PBMCs in culture cells by QIAzol method, and miRNAs were determined using two-step RT-qPCR with RT-primer specific assay in combination with TaqMan probes. The expression of miRNAs in PBMCs was performed by RT-qPCR using U6 as reference, miRNAs’ relative concentrations were normalized with Ct values of U6, and values were calculated using 2^−ΔΔCt^ and 2^−ΔCt^ formulas. The evaluations carried out were cells with RPMI-1640 cell medium (Control), treated with 100 µM NTZ, stimulated with anti-CD3 and anti-CD28 antibodies, and Abs + NTZ. The scattered dot plot shows individual measurements, and the bar represents the mean. Friedman’s test for paired samples *p* < 0.05, Tukey’s DHS post hoc, and exact *p* values for individual tests are shown.

PBMCs treated with NTZ showed lower relative levels of miR-155-5p than unstimulated cells (control) (*p* < 0.0001). Conversely, PBMCs stimulated with antibodies showed higher levels of miR-155-5p expression as compared with the untreated cells (control) (*p* = 0.0032). Moreover, the downregulation of the relative expression of miR-155-5p was found in cells treated with antibodies plus NTZ (Abs + NTZ) as compared with PBMCs stimulated only with antibodies (*p* < 0.0001). The relative expression of miR-146a-5p did not show any differences between all evaluated conditions (Figure 4).

### 3.5. NTZ Lowers the M1/M2 Ratio in PBMCs Stimulated with Anti-CD3 and Anti-CD28

**Figure 5:**
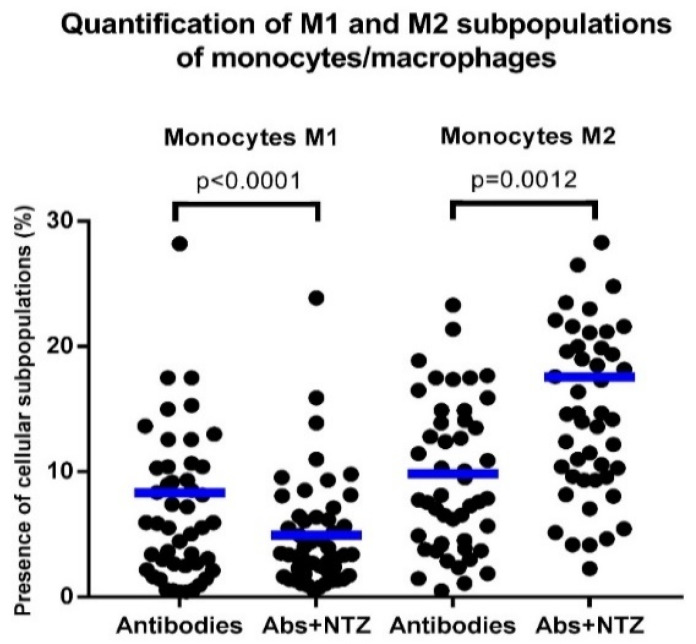
NTZ modifies the abundance M1 and M2 monocytes/macrophages. The quantification of proinflammatory M1 macrophages (CD14^+^, CD16^+^, and CD163^−^) and anti-inflammatory M2 macrophages (CD14^+^, CD16^+^, and CD163^+^) was made after 5 days of cell culture according to the type of treatment carried out on the PBMCs: cells stimulated with anti-CD3 and anti-CD28 antibodies and Abs + NTZ. The scattered dot plot shows individual measurements, and the bar represents the mean. Friedman’s test for paired samples *p* < 0.05, Tukey’s DHS post hoc, and exact *p* values for individual tests are shown.

**Figure 6:**
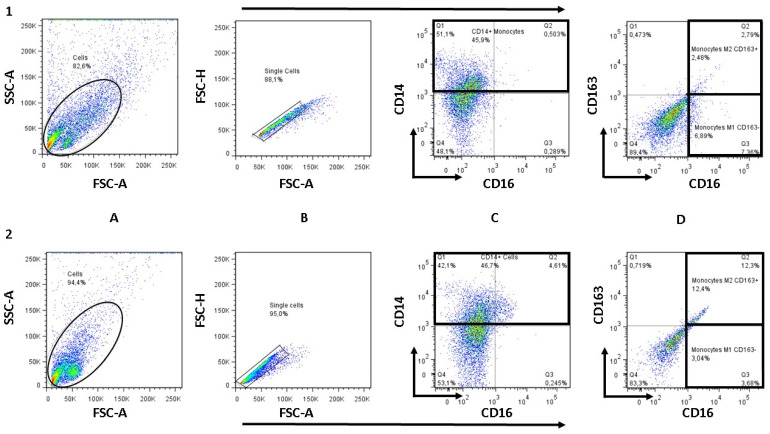
Strategy for the determination of monocytes/macrophages M1 and M2 by flow cytometry. (**A**) FSC (A) vs. SSC (A) plot: cells on forward scatter area/side scatter area plot. (**B**) FSC (A) vs. FSC (H): gating the cells that have an equal area and height: doublet exclusion gate. (**C**) CD16 vs. CD14 plot: gating to select monocytes based on their markers: selection of the CD14^+^ and CD16^+^ subpopulation gate. (**D**) CD16 vs. CD163 plot: gating to determine and quantify M1 (CD14^+^, CD16^+^, CD163^−^) and M2 (CD14^+^, CD16^+^, CD163^+^) monocytes/macrophages. For (**A**–**D**), the color represents cell density: from high density (red) to low density (blue). Sequence above: cells stimulated with anti-CD3 and anti-CD28 antibodies (Antibodies). Sequence below: cells stimulated with anti-CD3 and anti-CD28 antibodies and treated with 100 µM NTZ (Abs + NTZ).

PBMCs stimulated with antibodies and treated with NTZ (Abs + NTZ) showed lower proinflammatory M1 monocytes/macrophages than cells stimulated only with antibodies (*p* = 0.005), in addition to a concomitant increase in those anti-inflammatory M2 monocytes/macrophages (*p* = 0.007), resulting in a significant decrease in the M1/M2 ratio (Abs vs. Abs + NTZ, *p* < 0.001) (Figure 5 and Figure 6).

Regarding glycemic control, no significant changes were observed between controlled and uncontrolled patients with respect to the variables evaluated in this research.

## 4. Discussion

**Figure 7:**
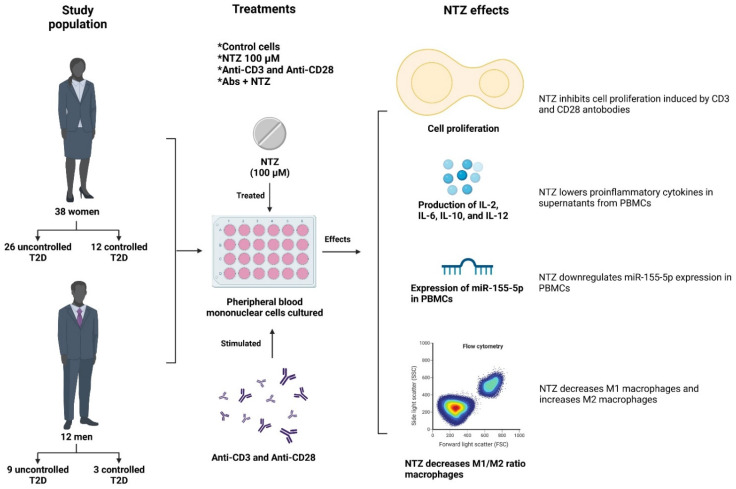
Nitazoxanide exerts immunomodulatory effects on peripheral blood mononuclear cells from type 2 diabetes patients. Fifty consecutive, randomized, and willing-to-participle patients with a clinical diagnosis of T2D without complications were selected [38]. Cells without monoclonal antibodies stimulation and without treatment with NTZ formed the control treatment; cells treated with 100 µM NTZ formed the NTZ treatment; cells stimulated with anti-CD3/anti-CD28 antibodies formed the Antibodies treatment, cells stimulated with anti-CD3/anti-CD28 antibodies treated with 100 µM NTZ formed the Abs + NTZ treatment. NTZ exerts an inhibitory effect on the cell proliferation of T cells stimulated with anti-CD3 and anti-CD28 antibodies without modifying cell viability and significant decreases in the supernatant concentrations of cytokines IL-2, IL-6, IL-10, and IL-12. Furthermore, NTZ negatively regulates the relative expression of miR-155-5p without changes in miR-146a-5p. The M1/M2 ratio of monocytes/macrophages decreased the M1 and increased the M2 subpopulation by NTZ.

In the current study, we found evidence for repurposing NTZ as an immunomodulatory drug because it has been shown to exert immunomodulatory effects in PBMCs of patients with T2D which show high proinflammatory cytokines [42], as well as increase circulating proinflammatory T lymphocytes and alterations in their proliferative capacity as compared with healthy subjects [43]. Our main findings are that NTZ inhibited cell proliferation induced by anti-CD3 and anti-CD28 antibodies, reduced the concentration of proinflammatory cytokines in cell culture supernatants, negatively regulated the expression of miR-155-5p without causing changes in miR-146a-5p concentration levels, and decreased the M1/M2 ratio of monocytes/macrophages (Figure 7). It is important to mention that the selection of subjects for this study included consecutive, randomized, and willing-to-participate patients. It has been reported that women have a greater concern for their medical care, which led to significantly greater use of health services and visits to clinics and primary care services for diagnosis as compared with men [44,45], which would explain why considerably more women than men participated in the study.

Although treatment with NTZ did not induce cytotoxic effects on PBMCs, it induced an inhibitory effect on cell proliferation. Cell viability and cell toxicity assays are both important tools for evaluating cellular responses to experimental compounds of interest [46,47]. Our results reinforce the findings described by Giacometti et al., who reported the absence of cytotoxicity by using different concentrations (1.6, 6.5, and 26 mmol/L) of NTZ (cytotoxicity from 8.9 to 11.2%) and NTZ together with antibiotics (cytotoxicity from 6.5 to 8.4%) in A-549 cell cultures [48]. Another study focused on evaluating NTZ cytotoxicity in cell lines of human glioblastoma (LN229, A172, and U87) and reported that concentrations of 1–20 μg/mL resulted in cell viability greater than 90% [33]. Furthermore, it has been described that exposure to NTZ (100 μM) in cultured HepG 2.2.15 cells did not induce cytotoxicity [49], indicating that this immunomodulatory effect of NTZ could be of clinical relevance. In our study, the immunomodulatory effect of NTZ was observed with a significant decrease in IL-2 levels with or without the stimuli of antibodies. This was expected because IL-2 is produced by activated T lymphocytes and is essential for growth, proliferation, differentiation, and survival of T cells. IL-2 is also crucial for the production of IL-6 for maintenance of the inflammatory process, inducing other cytokines synthesis also involved in T lymphocytes proliferation, independently of the expression/secretion of the IL-2 gene [50]. Our findings also show that NTZ produced a significant decrease in the level of IL-6 supernatants from cultured PBMCs, while the concentration of IL-6 was re-established with antibodies.

IL-6 is a pleiotropic cytokine that is considered to be a biomarker of inflammation [51] and a therapeutic target for the treatment of T2D [52]; thus, it is possible that IL-6 may act in a similar way to IL-12, determining the differentiation of naive CD4 T cells toward effector cells [53]. NTZ has also been shown to limit increased IL-12 production induced with or without antibody stimulation. IL-6 deficiency has been reported to lead to IL-12 depletion, resulting in a reduction in the inflammatory response, necessary to provide proliferation and survival signals to murine CD4 + T cells through the phosphatidylinositol 3-kinase/Akt signaling pathway [54], which could explain the effect of NTZ on both cytokines [55]. In addition, IL-1β is required as a T-cell-stimulating molecule for its activation and inflammation induction, although the mechanism has not yet been elucidated [56]. Our study found that NTZ reduced IL-1β secretion in cell-culture PBMCs. With respect to anti-inflammatory cytokines, NTZ did not modify IL-4 levels, while it re-established IL-10 baseline levels in the antibody-stimulated PBMCs. Thus, it suggested that NTZ was not involved in Th2 polarization because NTZ did not modify IL-4 production by exposed PBMCs, possibly due to its inhibitory effects on IL-6 synthesis, depressed by the effects of NTZ. However, previous studies had demonstrated that IL-6 induced an upregulation of IL-4 after short preincubations (5 min) of T cells stimulated with anti-CD3/anti-CD28, whereas with longer preincubation periods with IL-6 (12 and 24 h), the effect on IL-4 production gradually disappeared [57], without inducing alterations in IL-4 levels, reinforcing the results obtained in this study.

It is worth mentioning that the IL-6 levels in the supernatant of antibody-stimulated PBMCs correlated positively with BMI (r = 0.363, *p* = 0.001) and with the waist/hip ratio (r = 0.252, *p* = 0.007). These results also support the notion that the secretion of IL-6 in obesity is proportional to the expansion of fat mass [58]. The immunomodulatory effects of NTZ on cytokine concentrations are relevant because individual cytokine modulators are frequently used for severe inflammatory diseases, such as tocilizumab (IL-6 inhibitor) in rheumatoid arthritis [59], daclizumab (IL-2 inhibitor) in multiple sclerosis [60], and anakinra (IL-1 inhibitor) in other autoimmune diseases [61]. According to our findings, NTZ could become a potential candidate for anti-inflammatory treatment.

T cells have important functions in an acquired immune response; miRNAs regulate this immune response by targeting the miRNAs of genes involved in T cell development, proliferation, differentiation, and function. The miRNA expression changes during T cell activation highlight that their function can be constrained by a specific spatiotemporal frame related to the signals that induce T-cell-based effector functions [41]. T cell activation is dependent on TCR and co-stimulatory molecules (such as CD28). After that, T cells proliferate vigorously, leading to clonal expansion; miR-155-5p and miR-146a-5p are involved in the regulation of T cell proliferation. Our findings show that NTZ significantly decreased the concentration of miR-155-5p expression in cultured PBMCs, while diminished at baseline, increased levels resulted when cells were stimulated with antibodies, suggesting a transcriptional activity of NTZ in the NF-κB response. It is well known that miR-155-5p and miR-146a-5p are transcriptionally regulated by NF-κB [62] and coordinate inflammation, resulting in a defined inflammatory response [21]. We also found that NTZ treatment did not modify the expression of miR-146a-5p. Recently, studies have reported a significant decrease in miR-146a-5p expression as well as an increase in miR-155-5p expression in the blood serum of patients with T2D [63,64], causing loss in the suppressive effects of the Treg cells as a consequence of STAT1 overexpression [65] without repression in the expression of IRAK1 and TRAF6, which are adapter kinases of essential pathways such as MyD88 [66] for the maintenance of inflammation. The results obtained in this study confirm the overexpression of miR-155-5p in T2D and that NTZ could modulate the effects of miR-155-5p in PBMCs by repressing inositol-polyphosphate 5-phosphatase1 (SHIP1) or via the suppressor of cytokine signaling 1 (SOCS1), attenuating the activity of NF-κB and avoiding a proliferative state, which are crucial in the development of the immune response [67] and in the synthesis of immune effector molecules, e.g., cytokines. However, the molecular targets of NTZ related to the expression of miR-155-5p have not yet been elucidated.

Several studies have provided compelling evidence that microRNAs play pivotal roles in modulating macrophage activation, polarization, tissue infiltration, and resolution of inflammation and form a complex network that exerts widespread regulation of inflammatory pathways by targeting multiple components of the TLR signaling pathway and thus affects the profile of downstream-induced inflammatory cytokines [68]. The abovementioned studies have shown that an increase in the expression of miR-155-5p promoted polarization toward M1 macrophages while that of miR-146a-5p induced polarization toward M2 macrophages in murine and human by targeting various transcription factors and adapter proteins involved in IRF/STAT pathways [69]. This is important because treatments that act on miRNAs and that modulate macrophage polarization may have therapeutic potential in the treatment of inflammatory diseases. For the determination of M1 and M2 monocyte/macrophage subpopulations, CD163 can be used as a differential marker, which is expressed on the cell surface of activated monocytes/macrophages, exerting anti-inflammatory functions such as IL-10 production [70,71], and is cleaved from its surface in response to inflammatory stimuli (M1 macrophages) [72], suggesting that it could be used as a biomarker of meta-inflammation [73]. Interestingly, NTZ likely exerts immunomodulatory effects by decreasing the M1 proinflammatory subpopulation and increasing the M2 anti-inflammatory subpopulation of monocytes/macrophages, influencing, importantly, the M1/M2 ratio balance in favor of limiting the inflammatory process developed in T2D. Related to this result, peripheral blood monocytes from obese patients with T2D, as reported previously, were less susceptible to differentiating into an alternative phenotype, therefore contributing to a dysregulated and persistent inflammatory condition [74]. Our study revealed that NTZ could regulate the differentiation and activity of the M1 and M2 macrophages. The above is important because the development of M2 macrophages depends on the STAT-6 transcription factor [75], favoring glucose tolerance and reducing meta-inflammation [76], possibly being a therapeutic target of NTZ, and therefore more related studies are needed.

NTZ has been proposed to be an agonist of PPAR-γ receptors, with agonistic post-transcriptional effects capable of improving the glycemic profile in T2D [29]. Previously, Diana Kovács et al. (2013) used the unidimensional drug profile matching (oDPM) method to find possible PPARγ agonists [77]. It is worth mentioning that it is also possible that additional independent mechanisms of PPARs allow NTZ to exert its immunomodulatory effects.

In addition, the pharmacokinetics of NTZ reveal that its metabolite could reach a Cmax of 1.9 mg/L (7.1 mM) [27,78]; therefore, the 0.1 mM concentration used in our experiment could be easily reachable in the blood following drug administration, a result that is relevant for the evaluation of its effect as an immunomodulatory agent in in vivo models.

## 5. Conclusions

NTZ is positioned as a new approach for the treatment of T2D by regulating important immune functions. Our findings reveal the potential immunomodulatory and anti-inflammatory effects of NTZ by inhibiting cell proliferation, significantly reducing the serum levels of proinflammatory cytokines, decreasing the expression of miR-155-5p, and regulating the M1/M2 ratio balance in monocytes/macrophages. However, more studies are needed to reveal additional immunomodulatory effects of NTZ as well as its potential targets. These findings suggest alternative therapeutic benefits that could contribute to the future repositioning of NTZ, given the importance of the necessity for an immune-regulatory and adjuvant drug for treating T2D and other chronic and inflammatory diseases.

## Data Availability

The data presented in this study are available on request from the corresponding author.
